# RUNX2 interacts with SCD1 and activates Wnt/β‐catenin signaling pathway to promote the progression of clear cell renal cell carcinoma

**DOI:** 10.1002/cam4.5326

**Published:** 2022-10-06

**Authors:** Xiandong Song, Junlong Liu, Bitian Liu, Chiyuan Piao, Chuize Kong, Zhenhua Li

**Affiliations:** ^1^ Department of Urology The First Hospital of China Medical University Shenyang Liaoning P. R. China; ^2^ Department of Urology Shengjing Hospital of China Medical University Shenyang Liaoning P. R. China

**Keywords:** clear cell renal cell carcinoma, progression, runt‐related transcription factor 2, stearyl CoA desaturase 1, Wnt/β‐catenin

## Abstract

**Background:**

Previous studies have demonstrated that Runt‐associated transcription factor 2 (RUNX2) serves as the main transcription factor for osteoblast differentiation and chondrocyte maturation. RUNX2 is related to a variety of tumors, particularly tumor invasion and metastasis, while the expression and molecular mechanisms of RUNX2 in clear cell renal cell carcinoma (ccRCC) keep to be determined. Stearyl CoA desaturase 1 (SCD1), an endoplasmic reticulum fatty acid desaturase, transfers saturated fatty acids to monounsaturated fatty acids, is expressed highly in numerous malignancies.

**Methods:**

The Cancer Genome Atlas (TCGA) datebase and Western blot was used to analyzed the mRNA and protein levels of the target gene in ccRCC tissues and adjacent tissues. The proliferation ability of ccRCC cells was tested by colony forming and EdU assay. The migration ability of cells was detected by transwell assay. Immunoprecipitation was utilized to detect protein–protein interaction. Cycloheximide chase assay was used to measure the half‐life of SCD1 protein.

**Results:**

In this study, the expressions of RUNX2 and SCD1 are increased in ccRCC tissues as well as ccRCC cell lines. Both RUNX2 and SCD1 could promote proliferation and migration in ccRCC cells. Furthermore, RUNX2 could physically interact with SCD1. In addition, the functional degradation and the inactivation of Wnt/β‐catenin signaling pathway triggered by the downregulation of RUNX2 could be partly offset by the overexpression of SCD1.

**Conclusion:**

The findings indicate that the RUNX2/SCD1 axis may act as a potential therapeutic target via the Wnt/β‐catenin signaling pathway of ccRCC.

**Abbreviations**
ccRCC: clear cell Renal Cell Carcinoma
RUNX2: Runt‐related transcription factor 2
SCD1: Stearyl CoA desaturase 1
TCGA: The Cancer Genome Atlas

## 1 INTRODUCTION

Renal cell carcinoma (RCC) is considered a kind of common malignant tumor in the urinary system, after bladder cancer and prostate cancer in the aspect of incidence, with a proportion of 4.18% in whole adult malignancy and 21.82% in urinary malignancy.^1^, ^2^ RCC accounts for about 300,000 new cancer cases all over the world annually, contributing to almost 100,000 deaths every year.^3^ Clear cell renal cell carcinoma (ccRCC), as the most common histologic subtype, is a tumor regarded as originating from epithelial cells in the proximal convoluted tubule of the nephron.^4^ Eventually, around 50% of ccRCC patients would develop metastases. Although in the last few years the systemic treatment of metastatic RCC has come a long way, the patients' 5‐year survival rate with metastatic ccRCC turns out to be lower than 10%.^5^, ^6^ Thus, for improvement of the prognosis of patients with ccRCC, effective biomarkers along with brand‐new therapeutic targets are needed to recognize.

As a major transcriptional regulator of skeletogenesis, transcription factor Runt‐related transcription factor 2 (RUNX2) could regulate the expression of stage‐specific osteoblast genes and promote the transition from the immature to the mature osteoblast phenotype, thereby promoting bone formation.^7^, ^8^ Furthermore, the parts of RUNX2 played in migration, invasion, and metastasis have been recorded in different tumors including multiple myeloma, T‐cell lymphoma, acute myeloid leukemia, and prostate cancer.^9^, ^10^, ^11^, ^12^ According to the report, the expression of RUNX2 in tumors is at a higher level than that of normal tissue. Besides, the RUNX2 overexpression is significantly related to poor prognosis in various malignant cancers.^13^, ^14^ But, the pathophysiologic influence of RUNX2 on the progression of ccRCC is still undiscovered.

Lately, dysregulated fatty acid metabolism has been found among numerous kinds of cancers, for instance, breast cancer, lung cancer, prostate cancer, and ccRCC.^15^, ^16^, ^17^, ^18^ It has been demonstrated that aberrant lipid homeostasis is critical for sustained tumorigenesis in ccRCC.^19^ Stearyl CoA desaturase 1 (SCD1), as an endoplasmic reticulum fatty acid desaturase, is able to transform convert saturated fatty acids (SFAs) into monounsaturated fatty acids (MUFAs) during de novo synthesis of cellular fatty acids.^20^ Studies have revealed that the elevated expression of SCD1 could accelerate cell proliferation, enhance cell invasiveness, improve cell survival, and ultimately lead to greater tumorigenicity.^21^ SCD1 expression is evidently elevated in all types of human cancers, becoming a new key factor in tumorigenesis.^22^, ^23^ The study has been indicated that RUNX1 which is also a member of Runx family could regulate the expression of SCD1 in human skin squamous cell carcinoma. And Lipids and membrane organization are altered in response to RUNX1. Therefore, in this paper, we decided to detect the rationale that RUNX2 correlates with SCD1 in ccRCC.^24^

Abnormal activation in the Wnt/β‐catenin signaling pathway is taken as being able to increase the malignancy of human cancers of all kinds.^25^, ^26^ There is evidence that the Wnt/β‐catenin signaling pathway plays a dominant role in ccRCC growth together with metastasis.^27^

According to this research, it was revealed that RUNX2 and SCD1 were overexpressed in ccRCC, and RUNX2 might physically interact with SCD1. Moreover, RUNX2 might enhance the proliferation along with the migration of ccRCC via Wnt/β‐catenin signaling pathway activation.

## 2 MATERIALS AND METHODS

### 2.1 Bioinformatics analyses

For the purpose of accessing the mRNA expression levels of RUNX2 along with SCD1 in RCC tissues, the Cancer Genome Atlas (TCGA) was interrogated. Analysis of the TCGA platform is acquired by visiting the following website: http://gepia.cancer‐pku.cn (KIRC = clear cell renal cell carcinoma, KICH = chromophobe renal cell carcinoma, KIRP = papillary renal cell carcinoma).

### 2.2 Tissue samples

The tissue samples utilized in this research (tumor and normal tissues) came from 120 patients who were received and treated in the First Hospital of China Medical University (Shenyang, China) from November 2020 to February 2021. The tissue samples were frozen in liquid nitrogen at once when resection was done, then saved at −80°C. With the permission of the Ethics Committee of the First Hospital of China Medical University granted for the research, written informed consents were also achieved among every related patient.

### 2.3 Cell lines and cell culture

Each cell line, offered with 95% air and 5% CO_2_ at the temperature of 37°C_,_ was incubated in a humidified incubator. The human ccRCC cell lines, including 769‐P, 786‐O, and OS‐RC‐2, were incubated in an RPMI‐1640 medium (HyClone; GE Healthcare Life Sciences), and CAKI‐1 cells were incubated in McCoy's 5A medium (Gibco; Thermo Fisher Scientific, Inc.), and ACHN cells were incubated in an MEM medium (HyClone; GE Healthcare Life Sciences). HK‐2 cells (normal cortex/proximal tubule cells) were incubated in a DMEM/F12 medium (HyClone; GE Healthcare Life Sciences). Each medium was with supplementation of 10% fetal bovine serum (HyClone, USA). With regards to seeding and subcultivation, first, phosphate‐buffered saline (PBS) was used to wash cells and later digested by trypsin/EDTA solution until cells detached.

Cycloheximide (CHX) was purchased from MCE (MedChemExpress, USA). To determine the half‐life of SCD1 protein by CHX, cells were treated with CHX (100 μg/ml) for the indicated time and then harvested the cell protein for immunoblotting.

### 2.4 Western blot

RIPA buffer plus protease inhibitor cocktail was applied to lyse tissues and cells, and a BCA protein assay kit was employed for detecting the protein concentration. Equivalent protein was extracted electrophoretically by 10% SDS‐PAGE. Following an electrophoretic transfer of proteins onto PVDF membranes (Bio‐Rad Laboratories, Inc.), nonspecific binding was blocked by being incubated in 5% skim milk for 1 h at the temperature of 37°C, then the membranes were cultured at 4°C for the whole night by primary antibody as follows: anti‐RUNX2 (1:1000, 12,556, Cell Signaling Technology), anti‐SCD1(1:500, sc‐58,420, Santa Cruz Biotech), anti‐GSK‐3β (1:1000, D5C5Z, Cell Signaling Technology), anti‐P‐GSK‐3β (1:1000, D85E12, Cell Signaling Technology), anti‐AXIN1 (1:1000, C95H11, Cell Signaling Technology), anti‐DVL2 (1:1000, 30D2, Cell Signaling Technology), anti‐β‐tubulin (1:1000, 2128S, Cell Signaling Technology), or anti‐GAPDH (1:5000, 5174, Cell Signaling Technology). TBST (0.2% Tween) was used to wash the membranes thrice, 15 min for each time, and then the membranes were incubated with the proper horseradish peroxidase (HRP)‐conjugated secondary antibodies for 1 h at 37°C. At last, the luminescence system (Bio‐Rad, CA, USA) and ECL luminescence reagent were utilized to detect (Absin Biotechnology, Shanghai, China). The densitometric values of every band were calculated by ImageJ software (version 1.51; National Institutes of Health).

### 2.5 Quantitative real‐time PCR

Total RNA was separated through the usage of TRIzol reagent (Takara Biotechnology, Dalian, China). A ThermoFisher Scientific NanoDrop ND‐100 was used to determine the quality and quantity of RNA. The mRNA was reverse transcribed for cDNA synthesis accessing PrimeScript RT Master Mix (Takara, Dalian). Real‐time quantitative PCR was accessed with the LightCycler™480 II system (Roche diagnostics, Switzerland). Relative quantification was performed by the method of 2^−ΔΔCT^ with GAPDH as the internal reference. The primers (forward and reverse, respectively) used were as follows: RUNX2 (5’‐GCGCATTCCTCATCCCAGTA‐3′ and 5’‐GGCTCAGGTAGGAGGGGTAA‐3′), SCD1 (5’‐TCTAGCTCCTATACCACCACCA‐3′ and 5′‐ TCGTCTCCAACTTATCTCCTCC‐3′), β‐catenin (5′‐ GGAGAACTGGTCGCCATCAAG −3′ and 5′‐ ACATTGGGTTCTCCTCGGACC ‐3′), and GAPDH (5’‐GGAGCGAGATCCCTCCAAAAT‐3′ and 5’‐GGCTGTTGTCATACTTCTCATGG‐3′).

### 2.6 Lentiviral transduction

Stable cell lines were made by lentivirus infection. The infected cells were chosen for at least four passages by putting 10 μg/ml puromycin into a growth medium. Lentiviral‐based plasmids for RUNX2 and SCD1 knockdown and those for RUNX2 and SCD1 overexpression were purchased from GeneChem (Shanghai, China). Stable RUNX2 or SCD1 knockdown ccRCC cell lines were generated using the lentivirus vector containing short hairpin (sh) RNAs targeting RUNX2 (shRNA‐RUNX2), SCD1 (shRNA‐SCD1), or negative control vector (shRNA‐Ctrl). Viral particles containing the full‐length fragment RUNX2 or SCD1 were used to generate overexpression ccRCC cell lines (LV‐RUNX2 or LV‐SCD1). The negative control vectors (LV‐Ctrl) were also generated. shRNA‐RUNX2 sequence: CCGGCTAGTGATTTAGGGCGCATTCTCGAGAATGCGCCCTAAATCACTGAGTTTTTG and shRNA‐SCD1 sequence: CCGGGCACATCAACTTCACCACATTCTCGAGAATGTGGTGAAGTTGATGTGCTTTTTG.

### 2.7 Co‐immunoprecipitation assay

For immunoprecipitation, RIPA buffer plus protease inhibitor cocktail was utilized to lyse cells, and centrifugation was done with cells at 12,000 *g* for 20 min at 4°C. Then, the supernatants were incubated with 10 μl anti‐RUNX2 antibody (Cell Signaling Technology), 10 μl of anti‐SCD1 antibody (Santa Cruz Biotech), or 1 μl of anti‐IgG antibody (negative control, Cell Signaling Technology) overnight at 4°C in rotation. Then put into 10 μl Pierce Protein A/G Magnetic Beads (Thermo Fisher Scientific) and rotated the mixture at 4°C for another 2 h. By means of mild lysis buffer washed the beads thrice, followed by adding protein loading buffer (5×) and denatured at a temperature of 100°C for 10 min. Next perform a western blot.

### 2.8 Chromatin immunoprecipitation assay

The chromatin immunoprecipitation assay was conducted by a SimpleChiP™ Enzymatic Chromatin IP kit (Cell Signaling Technology, Danvers, MA, USA). In short, cells were cross‐linked by 37% formaldehyde for 15 min at 37°C, used glycine to quench the cross‐linking reaction, and then gathered the cells. The cross‐linked chromatin was digested until a length of around 150–900 bp with the micrococcal nuclease added to the collected cells. The cross‐linked chromatin was then, respectively, incubated with 10 μl of anti‐RUNX2 antibody (Cell Signaling Technology), 1 μl of anti‐IgG antibody (negative control, Cell Signaling Technology), or 10 μl of anti‐histone H3 antibody (positive control, Cell Signaling Technology) overnight at 4°C in rotation and incubated with Protein A/G‐Sepharose for 2 h. Beads were then recovered by centrifugation and washed two times with ChIP Wash Buffer. The antibody/protein/DNA cross‐link complexes were reversed by heating at the temperature of 65°C for 2 h, and DNA Purification Kit was performed to purify the DNA (Cell Signaling Technology, Danvers, MA, USA). Purified DNA was scrutinized by RT‐qPCR with promoter‐specific primers (forward and reverse, respectively): SCD1 Primer 1 (5′‐ CCAGTCAACTCCTCGCACTT‐3′ and 5’‐AAGGCTAGAGCTGGCAACG‐3′), SCD1 primer 2 (5’‐CCATTGTTCGCAGGCGTACC‐3′ and 5′‐ ACATCTCCGTCCCGTCTTCC‐3′).

### 2.9 Colony formation assays

Cells that grew at 80% confluence were trypsinized and moved to a fresh medium in a single‐cell suspension. Cells were diluted properly to seed on 6‐well plates (500 cells per well). Cells shall be permitted to develop for 10 days prior to being stained with crystal violet solution. ImageJ software was used to quantify Colony areas.

### 2.10 Transwell assays

Cells were serum‐starved for 24 h and seeded 2 × 10^4^ cells in transwell inserts (8‐μm pore size, Corning). Cell suspensions were put into inserts involving 200 μl serum‐free media on the top as well as 600 μl media with 10% FBS at the base chamber. After that, keep cell suspensions incubated at 37°C for 48 h. By use of cotton swabs wiped off cells in the upper chamber gently. Took images with an optical microscope to analyze via Image J software.

### 2.11 5‐ethynyl‐2′‐deoxyuridine (EdU) proliferation assay

Cells were planted into 6‐well plates with EdU (BeyoClick™, EDU‐488, China) and put into the medium (1:1000) for labeling. After being labeled, took away the culture medium followed by EdU staining, and the cells were incubated for 30 min with a click reaction cocktail (Tris–HCl, pH 8.5, 100 mmol/L; CuSO4, 1 mmol/L; Apollo 488 fluorescent azide, 100 μmol/L; and ascorbic acid, 100 mmol/L) in the darkroom, at indoor temperature. The cells were washed twice with 1 × PBS. Prior to observation under a fluorescence microscope, nuclei were stained with Hoechst followed by mounting and imaging.

### 2.12 Statistical analysis

For all the bar graphs, all experimental data are presented as mean ± SEM as indicated. Mann–Whitney *U* test, Pearson Chi‐square test, Pearson correlativity analysis, as well as Student's *t* test were implemented based on the instruction. *p* value of <0.05 was regarded as evidence. GraphPad Prism 9 software was used to perform statistical analyses (LaJolla, CA, USA).

## 3 RESULTS

### 3.1 mRNA expression levels of RUNX2 and SCD1 in RCC from the TCGA database

**FIGURE 1:**
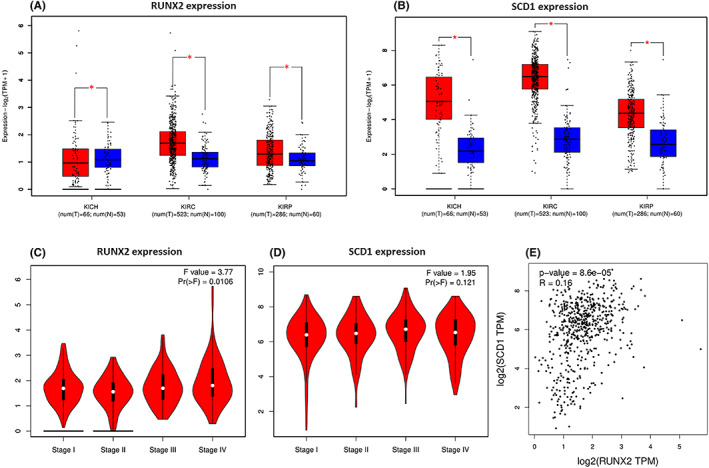
mRNA expression levels of RUNX2 and SCD1 in RCC from TCGA database. (A, B) RUNX2 was expressed at higher levels in KIRC and KIRP, whereas RUNX2 was downregulated in KICH. SCD1 was highly expressed in KICH, KIRC, and KIRP. (C, D) RUNX2 (left) or SCD1 (right) expression was not correlated with the pathological stage. (E) The mRNA expression of SCD1 was positively correlated to the mRNA expression of RUNX2 which is revealed by the TCGA platform at http://gepia.cancer‐pku.cn. KIRC, clear cell renal cell carcinoma; KICH, chromophobe renal cell carcinoma; KIRP, papillary renal cell carcinoma; red, tumor; blue, normal. *p* < 0.05 was considered significant, ns, not significant.

The expression of RUNX2 along with SCD1 in renal cell carcinoma via analyzing The Cancer Genome Atlas (TCGA) data sets was analyzed with the web link: http://gepia.cancer‐pku.cn. RUNX2 was upregulated in ccRCC and papillary renal cell carcinoma, and RUNX2 was downregulated in chromophobe renal cell carcinoma. SCD1 was upregulated in ccRCC, papillary renal cell carcinoma, and chromophobe renal cell carcinoma (Figure 1A, B). What is more, the expression of RUNX2 and SCD1 was not obviously correlated with the pathological stage in ccRCC (Figure 1C, D). TCGA platform revealed that SCD1 mRNA expression was in positive correlation with RUNX2 mRNA expression in ccRCC (Figure 1E).

### 3.2 RUNX2 and SCD1 were highly expressed in ccRCC tissues and ccRCC cell lines

**FIGURE 2:**
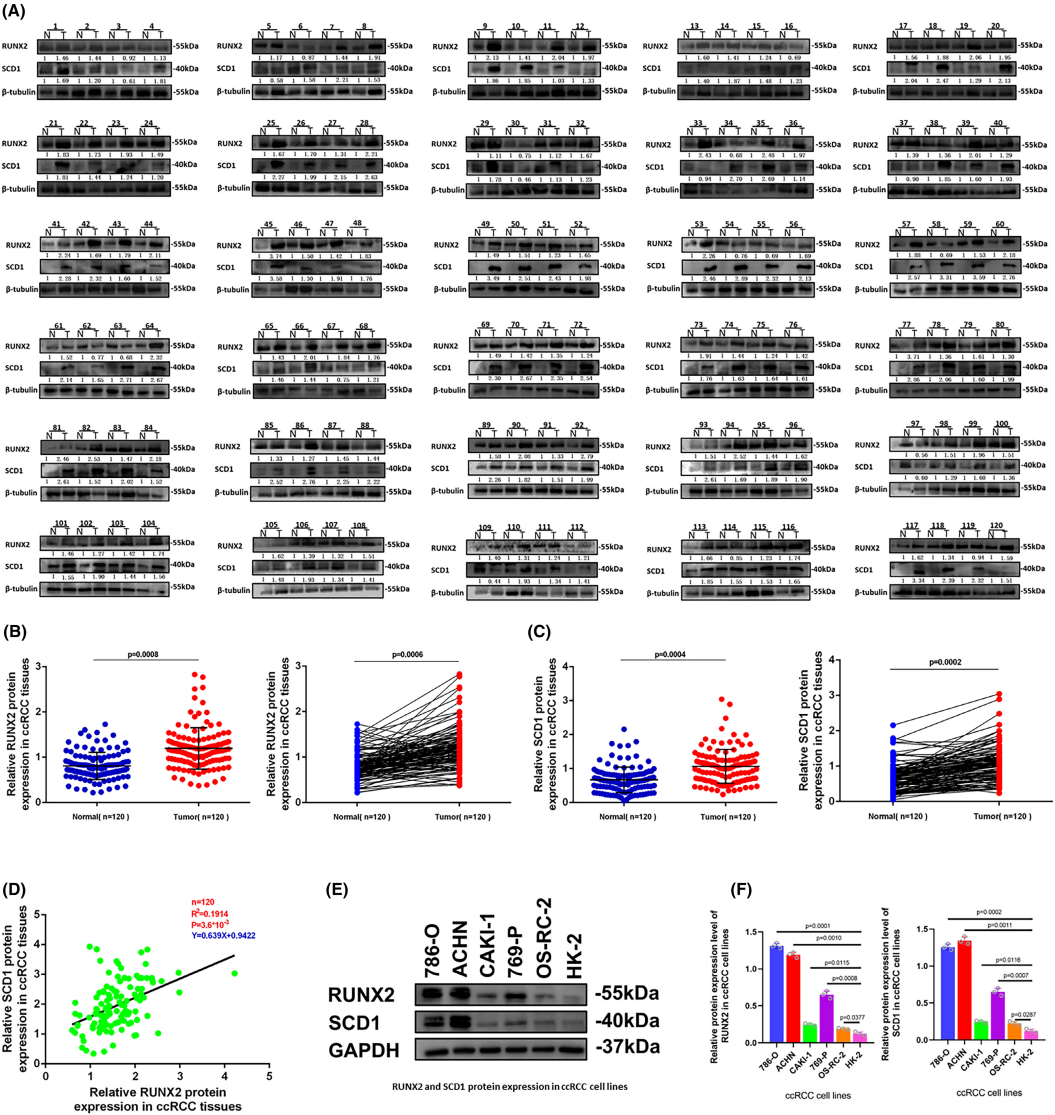
RUNX2 and SCD1 were expressed at high levels in ccRCC cell lines and ccRCC tissues. (A) RUNX2 and SCD1 protein levels in ccRCC tissues (T) and adjacent normal tissues (N). (B, C) RUNX2 and SCD1 were expressed at a significantly higher level in tumor tissues in comparison with normal tissues. (D) The protein expression of SCD1 was positively correlated to the protein expression of RUNX2 in ccRCC tissues. (E, F) The protein expression of RUNX2 was differently expressed among ccRCC cell lines. *p* < 0.05 was considered significant, ns, not significant.

RUNX2 and SCD1 expressions were analyzed in 120 pairs of ccRCC and adjacent normal tissues to explore potential clinical significance in ccRCC. As revealed by Western blot, the protein level of RUNX2 and SCD1 was prominently higher in tumor tissues than that in the adjacent normal tissues (Figure 2A–C). The expression of RUNX2 and SCD1 protein was significantly correlated with Fuhrman grade but failed to be remarkably related to age, gender, tumor stage, and tumor size (Table 1). According to this research, owing to the shortage of advanced tumors, the relationship with metastases could not be examined. Next, the interaction between the protein expression of RUNX2 and SCD1 in 120 cases of ccRCC was tested using linear correlation analysis, and the expression of RUNX2 was correlated positively with the protein level of SCD1 (Figure 2D). In addition, the protein expression level of RUNX2 was differentially expressed among different ccRCC cell lines (Figure 2E, F).

**TABLE 1:** Correlation between RUNX2 and SCD1 expression and the clinicopathological parameters of 120 ccRCC patients

			RUNX2			SCD1		
Variables	Group	N	Low	High	*p* value^a^	Low	High	*p* value^a^
Gender	Male	78	7	71	0.3743	9	69	0.3251
	Female	42	6	36		2	40	
Age	≥60	56	5	51	0.5712	6	50	0.7533
	<60	64	8	56		5	59	
T stage	T1/T2	108	12	96	>0.99	10	98	>0.99
	T3/T4	12	1	11		1	11	
Tumor size (cm)	≥5	30	1	29	0.1811	2	28	0.7289
	<5	90	12	78		9	81	
Fuhrman grade	1/2	111	9	102	0.0079*	7	104	0.0039*
	3/4	9	4	5		4	5	
Distant metastasis	Negative	117	12	105	0.2932	10	107	0.2525
	Positive	3	1	2		1	2	
Lymphatic invasion	Positive	0			–			–

^a^ *p* value was determined by Pearson Chi‐square tests and *P < 0.05 was considered significant.

### 3.3 RUNX2 played a crucial part in the proliferation and migration of ccRCC

**FIGURE 3:**
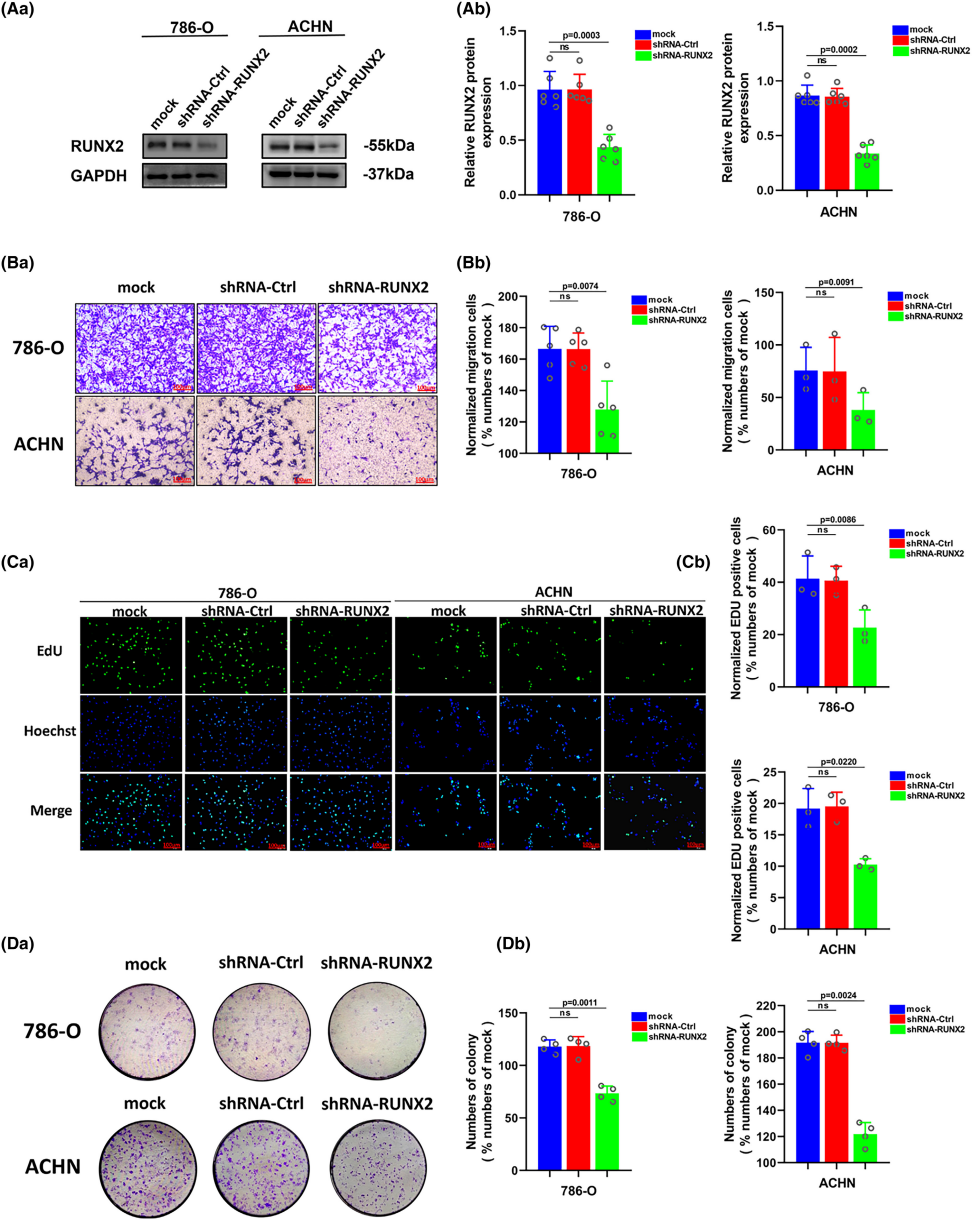
Downregulation of RUNX2 protein could inhibit proliferation and migration of ccRCC. (A) The protein level of RUNX2 was detected by western blot in 786‐O and ACHN cells transfected with shRNA‐RUNX2. (B) The migration ability of 786‐O and ACHN cells was detected by transwell assay (magnification×20). (C) The proliferation ability of 786‐O and ACHN cells was detected by EdU assay (magnification×200). (D) The colony formation ability of 786‐O and ACHN cells was detected by colony formation assay. *p* < 0.05 was considered significant, ns, not significant.

**FIGURE 4:**
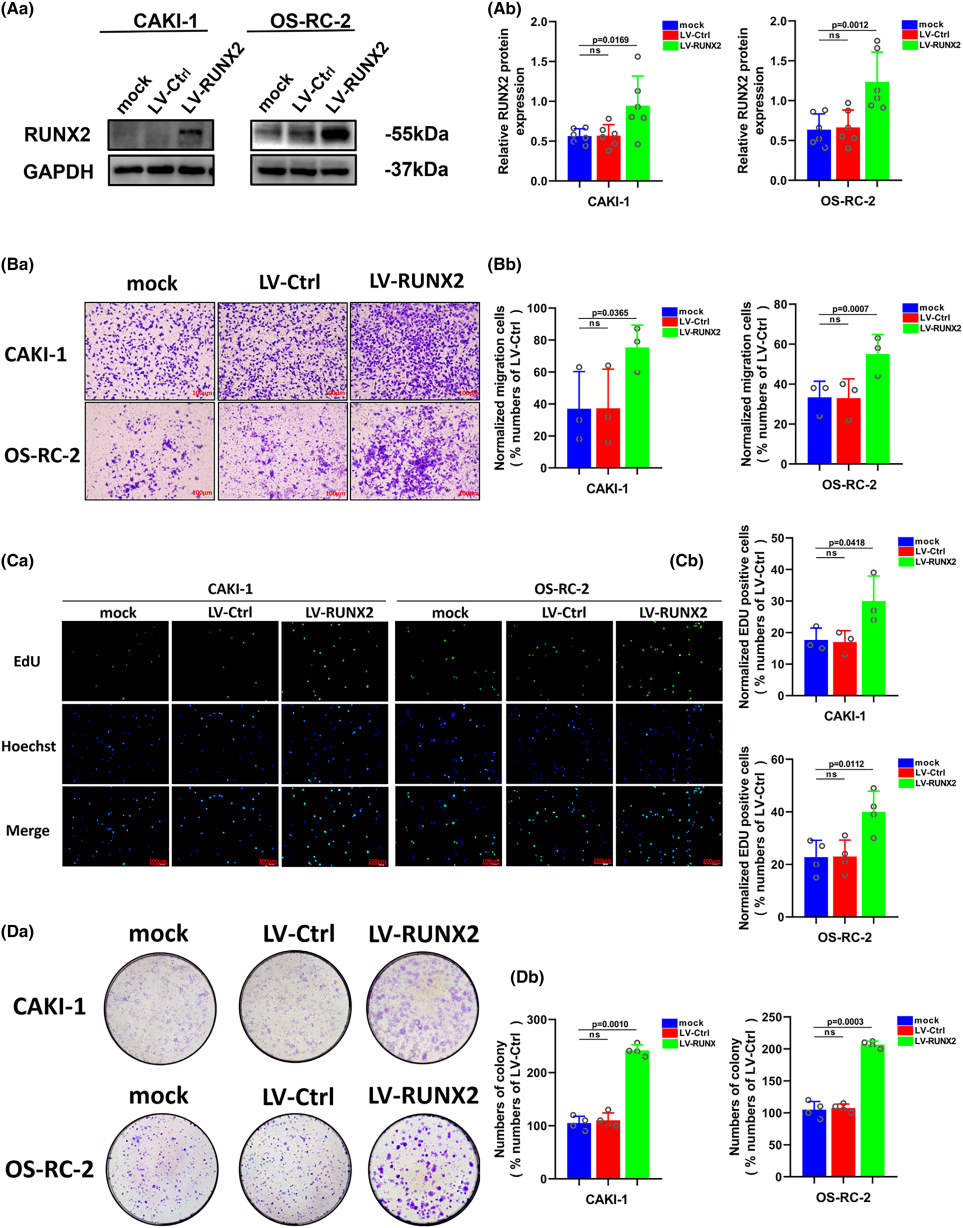
Upregulation of RUNX2 protein could promote proliferation and migration of ccRCC. (A) The protein level of RUNX2 was detected by western blot in CAKI‐1 and OS‐RC‐2 cells transfected with LV‐RUNX2. (B) The migration ability of CAKI‐1 and OS‐RC‐2 cells was detected by transwell assay (magnification×20). (C) The proliferation ability of CAKI‐1 and OS‐RC‐2 cells was detected by EdU assay (magnification×200). (D) The colony formation ability of CAKI‐1 and OS‐RC‐2 cells was detected by colony formation assay. *p* < 0.05 was considered significant, ns, not significant.

**FIGURE 5:**
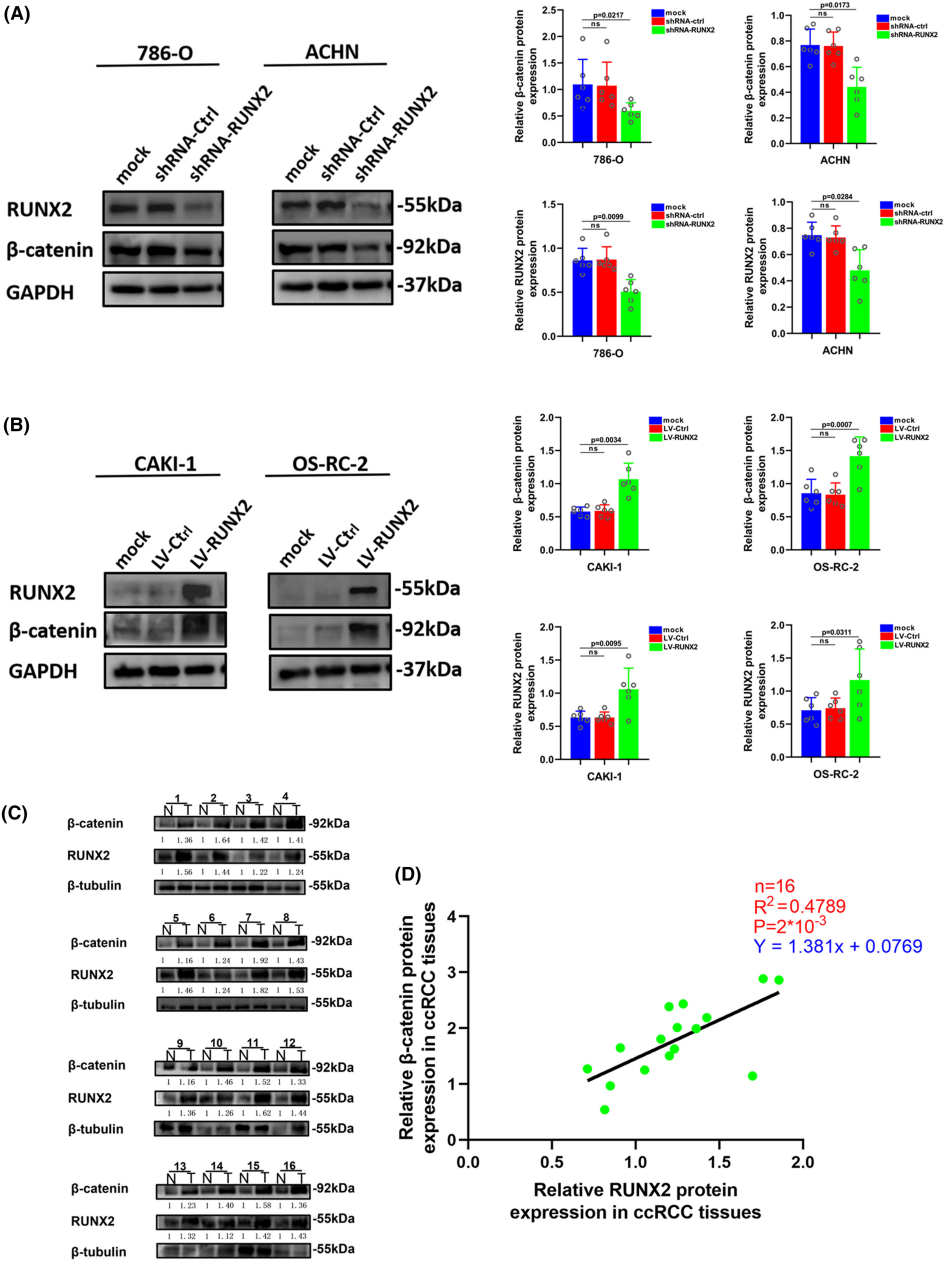
Wnt/β‐catenin pathway was activated by the overexpression of RUNX2. (A) The expression of β‐catenin was detected by western blot in 786‐O and ACHN cells transfected with shRNA‐RUNX2. (B) The expression of β‐catenin was detected by western blot in CAKI‐1 and OS‐RC‐2 cells transfected with LV‐RUNX2. (C) RUNX2 and β‐catenin protein levels in ccRCC tissues (T) and adjacent normal tissues (N). (D) The protein expression of β‐catenin was positively correlated to the protein expression of RUNX2 in ccRCC tissues. *p* < 0.05 was considered significant, ns, not significant.

Our previous study has provided evidence that RUNX2 could regulate epithelial–mesenchymal transition in renal cell carcinomas,^28^ and RUNX2 was found to mediate tumor growth and metastasis in clear cell renal cell carcinoma.^29^ Stable RUNX2 knockdown and RUNX2 overexpression ccRCC cell lines were generated to functionally dissect the potential role of RUNX2 in ccRCC cell growth and migration. The protein expression level of RUNX2 was greatly lowered owing to short hairpin (sh) RNAs targeting RUNX2 in 786‐O and ACHN cells (Figure 3A). Upregulation at the protein level of RUNX2 was observed in CAKI‐1 and OS‐RC‐2 cells transfected with viral particles containing the full‐length fragment of RUNX2 (Figure 4A). According to the migration assay, the migration capacity of 786‐O and ACHN cells was noticeably inhibited by the silence of RUNX2 while the migration ability of CAKI‐1 and OS‐RC‐2 cells was promoted as a result of RUNX2 overexpression (Figure 3B and 4B). EdU assay indicated that cellular growth was impaired by RUNX2 knockdown, and the proliferation ability of ccRCC cells was enhanced with RUNX2 overexpression (Figures 3C and 4C). Colony formation experiments revealed that the colony ability was impeded by depletion of RUNX2, whereas a significantly increased number of colonies were yielded when RUNX2 was overexpressed (Figure 3D and 4D). Given that the Wnt/β‐catenin signaling pathway holds the key to the progress of a variety of diseases and cancers and RUNX2 could control bone resorption through the downregulation of the Wnt/β‐catenin signaling pathway in osteoblasts,^30^, ^31^ the part of RUNX2 played in the activity of Wnt/β‐catenin signaling pathway was examined. As shown in Western blot, protein expression of β‐catenin was downregulated when RUNX2 was knocked down, and the expression of β‐catenin grew at the protein level when RUNX2 was upregulated (Figure 5A, B). Then the protein expression of RUNX2 and β‐catenin was analyzed in 16 pairs of ccRCC and adjacent normal tissues. As revealed by the western blot, RUNX2 and β‐catenin protein expression were higher in tumor tissues than that in the adjacent normal tissues (Figure 5C). Next, a linear correlation analysis was used to test the interaction between the protein expression of RUNX2 and SCD1 in 16 cases of ccRCC, and the expression of RUNX2 was correlated positively with the protein level of β‐catenin (Figure 5D). Collectively, these in vitro findings demonstrated that RUNX2 could promote the growth and migration of ccRCC cells by Wnt/β‐catenin signaling pathway activation. However, what is the rationale that RUNX2 could regulate Wnt/β‐catenin signaling? The mRNA expression level of RUNX2 and β‐catenin was then determined to explore the regulatory mechanism. It was found that when RUNX2 was knocked down, the mRNA expression level of RUNX2 was significantly declined, while there was no observed change in β‐catenin mRNA level (Figure S1). It could be conducted that RUNX2 does not regulate β‐catenin expression transcriptionally. Some studies have demonstrated that GSK‐3β, AXIN1, and DVL2 are the important regulators of Wnt/β‐catenin signaling.^32^, ^33^, ^34^ Therefore, we wondered if RUNX2 could activate Wnt/β‐catenin signaling by regulating GSK‐3β, AXIN1, and DVL2. As shown in (Figure S1), the expression of β‐catenin was significantly declined at the protein level in the absence of RUNX2. However, the protein expression of GSK‐3β, AXIN1, and DVL2 has no change. These data suggested that RUNX2 could activate Wnt/β‐catenin signaling but not via regulating GSK‐3β, AXIN1, and DVL2.

### 3.4 RUNX2 regulated the SCD1 expression in ccRCC cell lines

**FIGURE 6:**
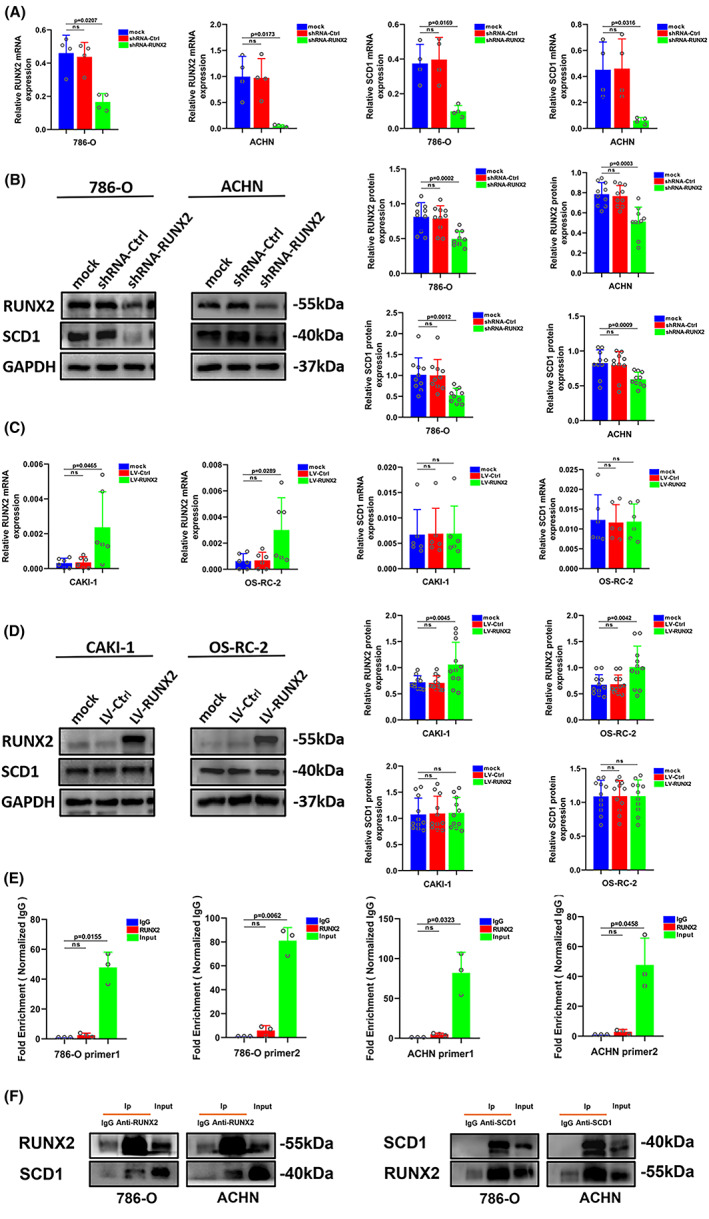
RUNX2 regulated the SCD1 protein expression in ccRCC cells. (A) The mRNA level of RUNX2 and SCD1 was determined by RT‐qPCR in 786‐O and ACHN cells transfected with shRNA‐RUNX2. (B) The protein level of RUNX2 and SCD1 was determined by western blot in 786‐O and ACHN cells transfected with shRNA‐RUNX2. (C) The mRNA level of RUNX2 and SCD1 was determined by RT‐qPCR in CAKI‐1 and OS‐RC‐2 cells transfected with LV‐RUNX2. (D) The protein level of RUNX2 and SCD1 was determined by western blot in CAKI‐1 and OS‐RC‐2 cells transfected with LV‐RUNX2. (E) The fold enrichment of RUNX2 on SCD1 promotor in 786‐O and ACHN cells with high RUNX2 expression. (F) The physical interaction between RUNX2 and SCD1 in 786‐O and ACHN cells was examined by co‐immunoprecipitation assay. *p* < 0.05 was considered significant, ns, not significant.

Stable RUNX2 knockdown and RUNX2 overexpression ccRCC cell lines were used to study whether RUNX2 could regulate SCD1 expression in ccRCC cells. In the absence of RUNX2, the expression of SCD1was significantly declined at the mRNA and protein level (Figure 6A, B), but no striking change was perceived in the mRNA and protein expression of SCD1 with the overexpression of RUNX2 in ccRCC cells (Figure 6C, D). Given that RUNX2 serves as a recognized transcription factor and SCD1 expression regulation is mediated by a variety of different transcription factors, notably sterol response element‐binding protein (SREBP), peroxisome proliferator‐activated receptor (PPAR), LXR, NF‐1, and AP‐2,^35^, ^36^, ^37^ chromatin immunoprecipitation assay was conducted. However, it was revealed that RUNX2 could not bind to the promoters of SCD1 (Figure 6E). Therefore, it could be hypothesized that the upregulation of RUNX2 expression could not affect the expression of SCD1, and there might be some co‐mediating factors or some other regulation modes between them. Co‐immunoprecipitation assay indicated that anti‐RUNX2 antibody could co‐immunoprecipitate with SCD1 in ccRCC cells. Similarly, RUNX2 was co‐immunoprecipitated using an anti‐SCD1 antibody (Figure 6F). Taking together, RUNX2 could physically interact with SCD1. The study has indicated that SCD1 protein could be degraded in the ubiquitin‐proteasome‐dependent pathway.^38^ The half‐life of the SCD1 protein was then analyzed by the cycloheximide chase assay. First, 786‐O cells were harvested with CHX (100 μg/ml) for 0, 3, 6, and 9 h. As the western blot is shown (Figure S2), the protein content of SCD1 at 3 h was degraded to about 70% of the protein content at 0 h, and at 6 h, the protein content was degraded to about 30% of the protein content of SCD1 at 0 h. Then, 786‐O cell was treated with CHX (100 μg/ml) for 0, 3, 4, 5, and 6 h. It was found that the protein content of SCD1 was degraded to about 50% at 5 h compared with that at 0 h (Figure S2). Therefore, it could be concluded that the half‐life of the SCD1 protein was 5 h. Subsequently, cells stably expressing shRNA‐Ctrl and shRNA‐RUNX2 were treated with CHX (100 μg/ml) for the indicated time, then harvested the cell protein for immunoblotting. The half‐life of the SCD1 protein was reduced after RUNX2 depletion (Figure S2). These results suggest that RUNX2 might regulate the stability of the SCD1 protein, probably by inhibiting its ubiquitination degradation pathway.

### 3.5 SCD1 partially restored the effect of RUNX2 in the progression of ccRCC

**FIGURE 7:**
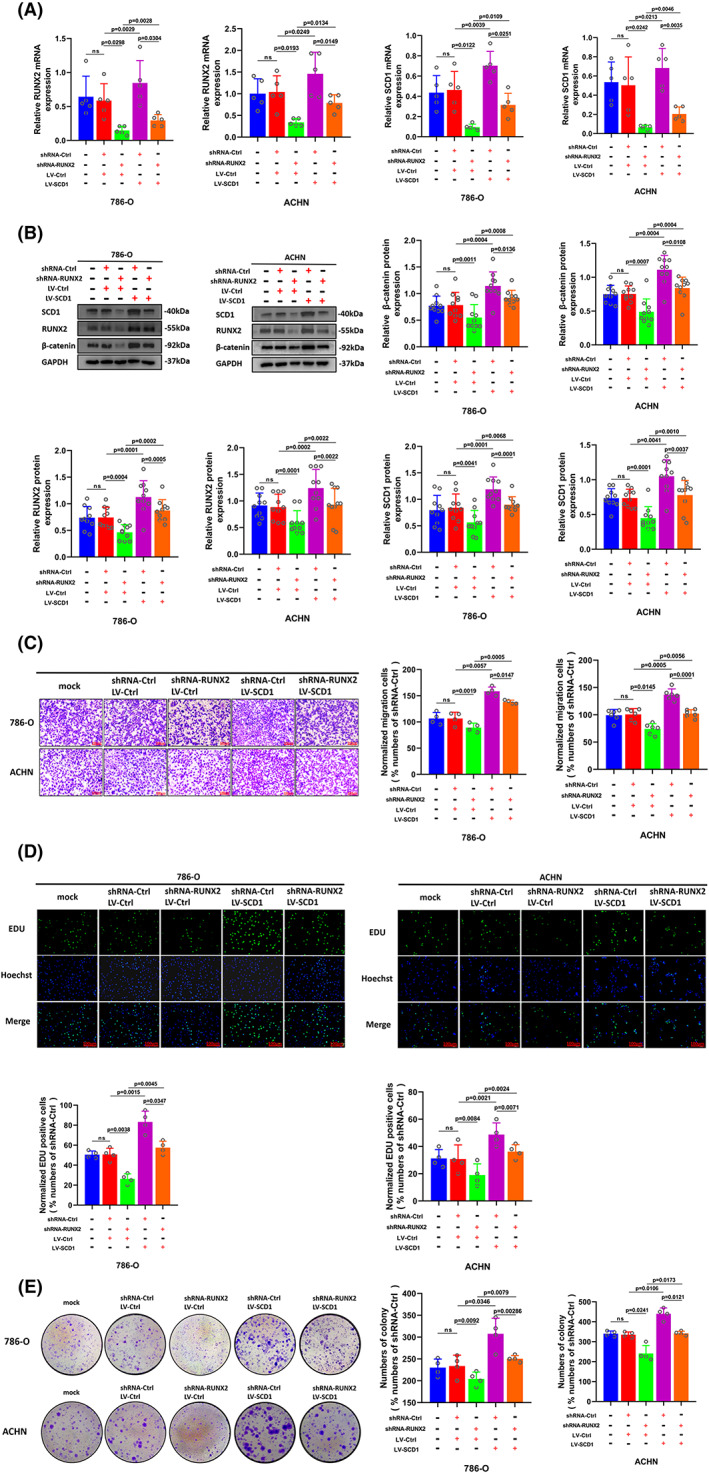
The decline of cell ability in proliferation and migration caused by RUNX2 knockdown was partially reversed with SCD1 overexpression. (A) Cells transfected with shRNA‐Ctrl and shRNA‐RUNX2 were transfected with LV‐Ctrl or LV‐SCD1, and the mRNA expression of RUNX2 and SCD1was determined by RT‐qPCR. (B) The protein expression of β‐catenin, RUNX2, and SCD1 was detected by Western blot. (C) The migration ability of 786‐O and ACHN cells was examined by transwell assay (magnification × 20). (D) The proliferation ability of 786‐O and ACHN cells was determined by EdU assay (magnification × 200). (E) The colony formation ability of 786‐O and ACHN cells was determined by colony formation assay. *p* < 0.05 was considered significant, ns, not significant.

**FIGURE 8:**
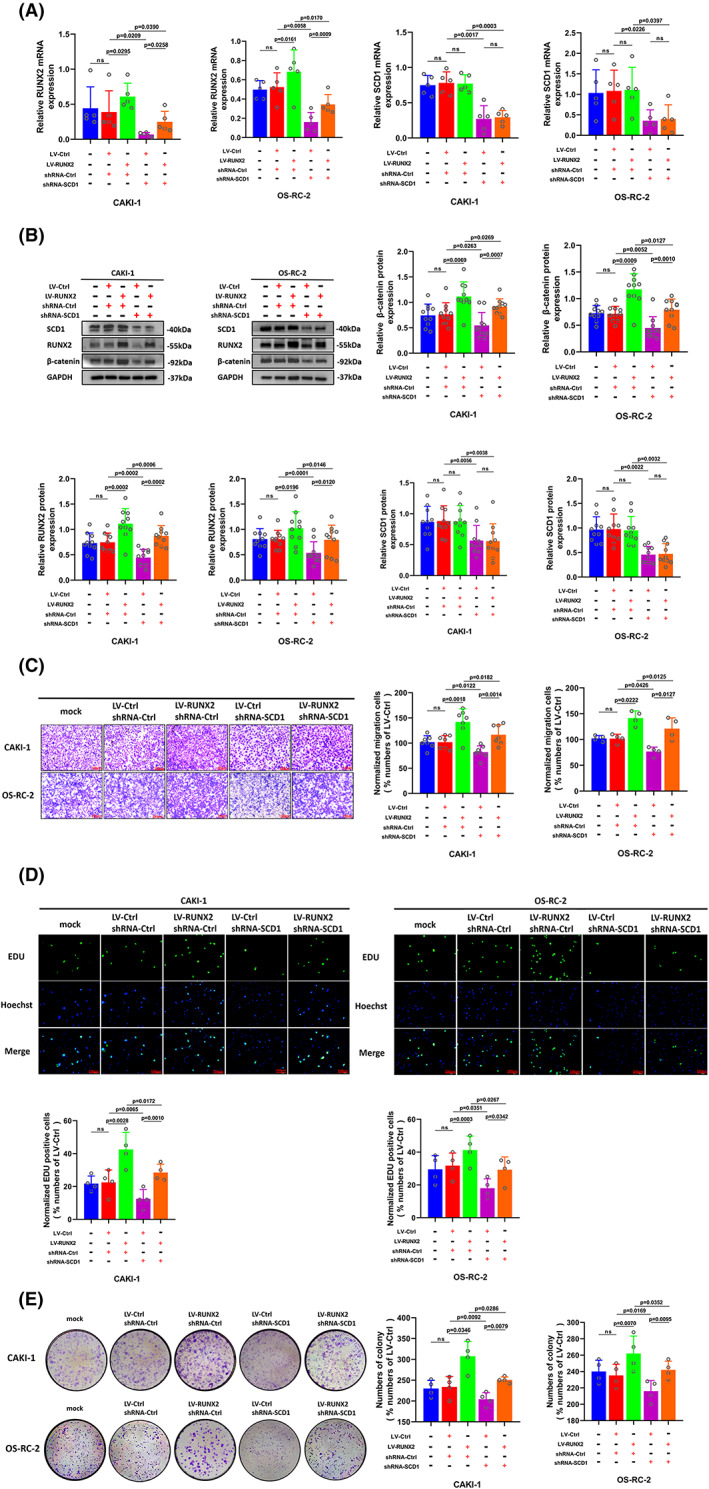
The elevated potential of cell proliferation and migration caused by RUNX2 overexpression was partially restored by the downregulation of SCD1. (A) Cells transfected with LV‐Ctrl and Lv‐RUNX2 were transfected with shRNA‐Ctrl or shRNA‐SCD1, and the mRNA expression of RUNX2 and SCD1was determined by RT‐qPCR. (B) The protein expression of β‐catenin, RUNX2, and SCD1 was detected by Western blot. (C) The migration ability of CAKI‐1 and OS‐RC‐2 cells was examined by transwell assay (magnification × 20). (D) The proliferation ability of CAKI‐1 and OS‐RC‐2 cells was determined by EdU assay (magnification × 200). (E) The colony formation ability of CAKI‐1 and OS‐RC‐2 cells was determined by colony formation assay. *p* < 0.05 was considered significant, ns, not significant.

For the sake of investigating the part of SCD1 played in RUNX2‐mediated ccRCC cell growth, migration, and invasion, the RUNX2 overexpression cells were transfected with short hairpin (sh) RNAs targeting SCD1 (LV‐RUNX2/shRNA‐SCD1) and the RUNX2 knockdown cells were transfected with viral particles containing the full‐length fragment of SCD1 (shRNA‐RUNX2/LV‐SCD1). The RUNX2 expression was elevated at the mRNA and protein level when SCD1 was overexpressed and the protein expression level of the β‐catenin signaling pathway also rose after overexpression of SCD1 (Figure 7A, B). The migration assay showed that the migration potential in RUNX2 knockdown ccRCC cells was enhanced as a result of SCD1 overexpression (Figure 7C). Additionally, EdU assay and colony formation assay revealed that ccRCC cell proliferation was impaired when deficiency of RUNX2, whereas the decline in cell growth resulted from the silence of RUNX2 was incompletely restored with SCD1 overexpression (Figure 7D, E). Conversely, the mRNA and protein expression of RUNX2 was downregulated with the silence of SCD1, and the expression of β‐catenin was also decreased at a protein level (Figure 8A, B). The enhanced ability of migration, proliferation as well as colony formation caused by RUNX2 overexpression was partially reversed by SCD1 knockdown (Figure 8C–E). These data suggested that the RUNX2‐SCD1 axis was likely to be conducive to the development of ccRCC.

## 4 DISCUSSION

In this research, RUNX2 was expressed at a higher level in ccRCC cell lines and ccRCC tissues by demonstration. Overexpression of RUNX2 was able to enhance ccRCC cell growth and migration as well as activate the Wnt/β‐catenin signaling pathway. Furthermore, this study indicates that RUNX2 might be a potential biomarker and a therapeutic target for the clinical treatment of ccRCC. Besides, RUNX2 could physically interact with SCD1, contributing to ccRCC cell proliferation and migration. Such data show that RUNX2 could intermodulate with SCD1 to accelerate the development of ccRCC by activating the Wnt/β‐catenin signaling pathway.

RUNX2 is thought to relate to the proliferation, invasion, and metastasis of diverse malignant tumor cells.^13^, ^39^, ^40^ Until now, RUNX2 expression has been mainly reported in cells of the osteoblast lineage, and RUNX2 plays a critical part as bone marrow mesenchymal stem cells (BMSCS) differentiate and mature into osteoblasts during the development of bone.^41^, ^42^, ^43^ Nevertheless, the effect of RUNX2 on ccRCC is open to clarification. From this research, RUNX2 was discovered to be highly expressed in both ccRCC cell lines and ccRCC tissues. RUNX2 was irrelevant to gender, age, tumor size, and tumor stage. Nevertheless, RUNX2 was markedly related to Fuhrman grade. A recent study suggests that the clinical survival rate of ccRCC patients with high expression of RUNX1 is lower than that of ccRCC patients embodied with low RUNX1 expression.^44^ These data reveal that RUNX2 might have a protumorigenic effect in ccRCC.

To functionally dissect the potential influence of RUNX2 on the pathogenesis of ccRCC, the expression of RUNX2 in ccRCC cell lines was examined. With regards to the expression of RUNX2, high levels in 786‐O and ACHN cells, moderate levels in 769‐P cells, and CAKI‐1 and OS‐RC‐2 cells at low levels were observed. The 786‐O, ACHN, CAKI‐1, and OS‐RC‐2 cells were selected for finding out the biological roles of RUNX2 in the invasion as well as proliferation of ccRCC cells. Given that dysregulation of the Wnt/β‐Catenin signaling pathway is also an independent predictor of oncologic results in patients with ccRCC,^45^ it was discovered that the clone formation, proliferation, and migration ability of ccRCC cells was weakened by RUNX2 knockdown and the Wnt/β‐catenin signaling pathway was also inactivated, while there was an opposite outcome when RUNX2 was upregulated. And the protein expression of RUNX2 and β‐catenin was upregulated in ccRCC tumor tissues. There was an obvious correlation between RUNX2 and β‐catenin expression at protein levels. Studies have demonstrated that RUNX2 could alleviate high glucose‐suppressed osteogenic differentiation via the Wnt/β‐catenin pathway and it has also been discovered that RUNX2 could control osteosarcoma apoptosis via the Wnt/β‐catenin signaling pathway.^46^, ^47^ It was demonstrated that GSK‐3β, AXIN1, and DVL2 could regulate the Wnt/β‐catenin signaling pathway.^48^, ^49^, ^50^ In our paper, it was revealed that the β‐catenin expression could not be regulated via RUNX2 transcriptionally at the mRNA level, and the protein expression of important regulators of the Wnt/β‐catenin signaling pathway such as GSK‐3β, AXIN1, and DVL2 was not affected by RUNX2 expression. It could be concluded that RUNX2 could activate Wnt/β‐catenin signaling but not via regulating GSK‐3β, AXIN1, and DVL2. In this study, it is demonstrated that RUNX2 might promote the development and progression of ccRCC via Wnt/β‐catenin signaling pathway activation. However, how RUNX2 could regulate Wnt/β‐catenin signaling. More experiments still need to be done in the future. SCD1 is a lipid‐modifying enzyme that catalyzes the ttransformation of saturated fatty acid to a monounsaturated fatty acid. Besides, the expression of SCD1 is commonly upregulated in diverse tumor types.^22^, ^51^, ^52^ Studies have demonstrated the involvement of SCD1 in the promotion of proliferation, migration, metastasis, and tumor growth in cancer cells of different origins including the kidneys, bladder, liver, colon, thyroid, and endometrium.^23^, ^53^, ^54^, ^55^, ^56^, ^57^ Western blot analysis of ccRCC tissues derived from clinical tumor specimens revealed that SCD1 protein expression was in positive correlation with RUNX2 protein expression. The mRNA and protein expression of SCD1 was decreased on account of the downregulation of RUNX2, whereas no notable change was found in SCD1 expression at the mRNA and protein level when RUNX2 was overexpressed. It could be inferred that there might be some unknown factor between RUNX2 and SCD1 that participated in the regulatory relationship between them. Chromatin immunoprecipitation assay revealed that RUNX2 could not bind to the promoters of SCD1, whereas the RUNX2 protein could interact with SCD1 protein physically, which was demonstrated by the co‐immunoprecipitation assay. Studies have indicated that SCD1 is ubiquitinylated prior to proteasomal degradation and there is supporting evidence illuminating that the 66‐residue N‐terminal segment primarily takes charge of SCD1 ubiquitin degradation and this segment induces instability in an otherwise stable endoplasmic reticulum membrane protein.^38^, ^58^ It could be hypothesized that the decrease of SCD1 may be induced by RUNX2 via the ubiquitin‐proteasome‐dependent pathway. Cycloheximide tracing experiment showed that the degradation rate of SCD1 protein was significantly accelerated in the absence of RUNX2 protein. In summary, RUNX2 might activate and maintain the stability of the SCD1 protein. Finally, it was found that the ability of ccRCC cells proliferation and migration was partially restored by a protein rescue experiment. A report has identified that the overexpression of SCD1 is in relation to the growth, migration, and invasion of numerous neoplastic lesions.^59^, ^60^ From this research, the mRNA and protein expression of RUNX2 was enhanced when SCD1 was overexpressed, and the RUNX2 expression was lowered at the mRNA and protein level as a result of SCD1 knockdown. Furthermore, in RUNX2 knockdown cells, the ccRCC progression was enhanced by SCD1 overexpression and the Wnt/β‐catenin signaling pathway was activated as SCD1 was overexpressed. Conversely, the migration and proliferation ability of ccRCC was inhibited by SCD1 silencing in RUNX2 overexpression cells and the activation of Wnt/β‐catenin signaling pathway was also decreased when SCD1 was knockdown. Therefore, it could be speculated that co‐expression of RUNX2 and SCD1 might play an essential role in ccRCC cell proliferation and migration by activating the Wnt/β‐catenin signaling pathway.

In conclusion, RUNX2 could intermodulate with SCD1 and activate the Wnt/β‐catenin signaling pathway to facilitate the progress of ccRCC. However, there might be some co‐mediating factors or some other regulatory modes between RUNX2 and SCD1. Consequently, it is in need of exploring the regulatory mechanism between them further.

## AUTHOR CONTRIBUTIONS

XS carried out experiments and also completed the manuscript; JL and BL gathered the data; CP carried out data analysis; and CK and ZL proceeded with experimental design and supervised all experiments and manuscripts. All related authors have examined and approved the final manuscript.

## Funding information

No particular grant that belonged to funding agencies in public, commercial, or nonprofit fields was given to such a study.

## CONFLICT OF INTEREST

The authors declare that the research was conducted in the absence of any commercial or financial relationships that could be construed as a potential conflict of interest.

## ETHICAL APPROVAL STATEMENT

With the permission of the Ethics Committee of the First Hospital of China Medical University granted for the research, written informed consents were also achieved among every related patient.

## Supporting information


Figure S1

Figure S2

## Data Availability

The data sets employed and/or analyzed in the current study are attainable from the relevant author at reasonable request.
