# Supplementary figures and images for: RUNX2 interacts with SCD1 and activates Wnt/β‐catenin signaling pathway to promote the progression of clear cell renal cell carcinoma
Source: Cancer Med. 2022 Oct 6;12(5):5764–80. doi: 10.1002/cam4.5326 (PMC10028032; doi:10.1002/cam4.5326)

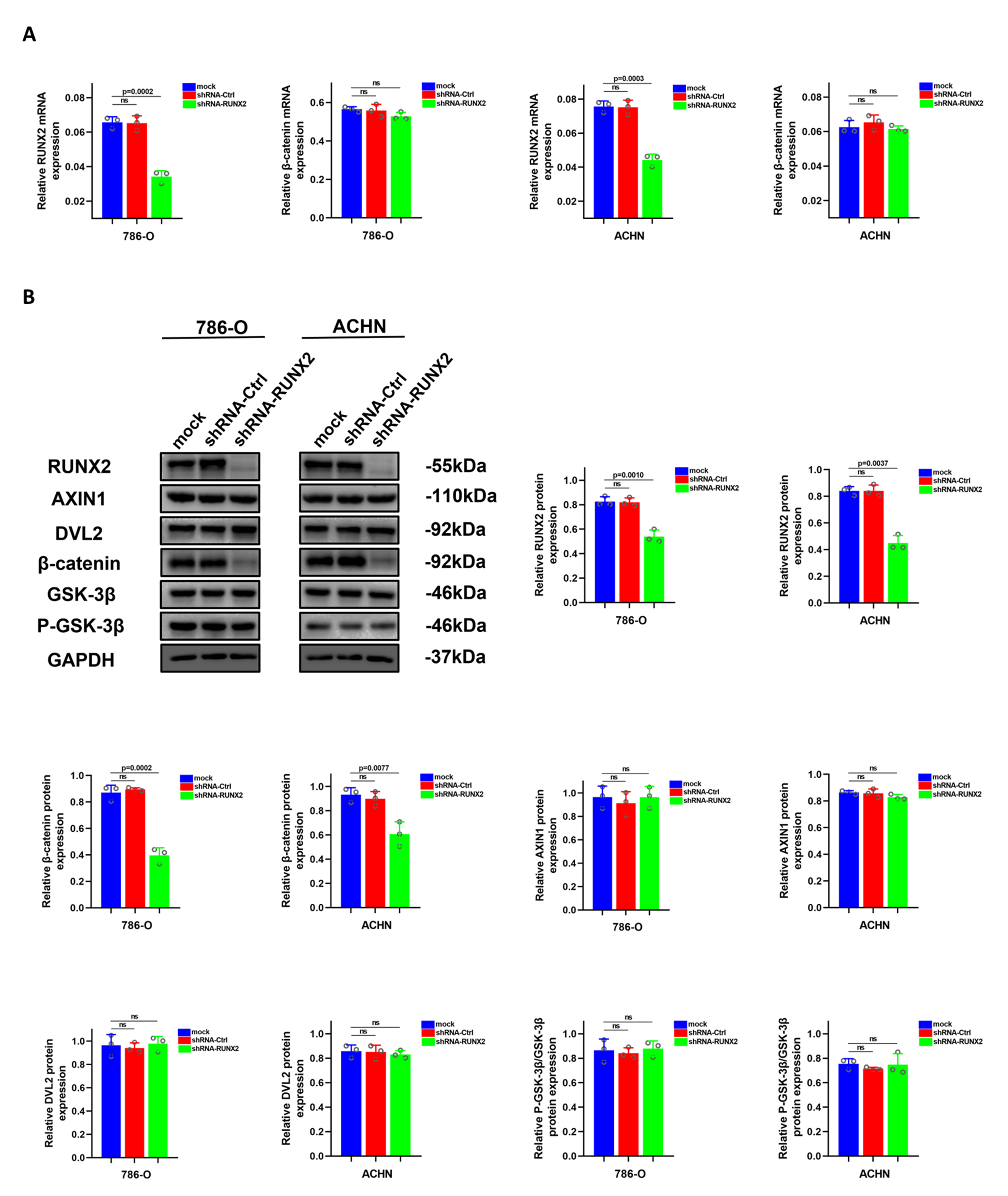

Supplement: Supplementary file 1 — Figure S1 [file CAM4-12-5764-s001.tif]

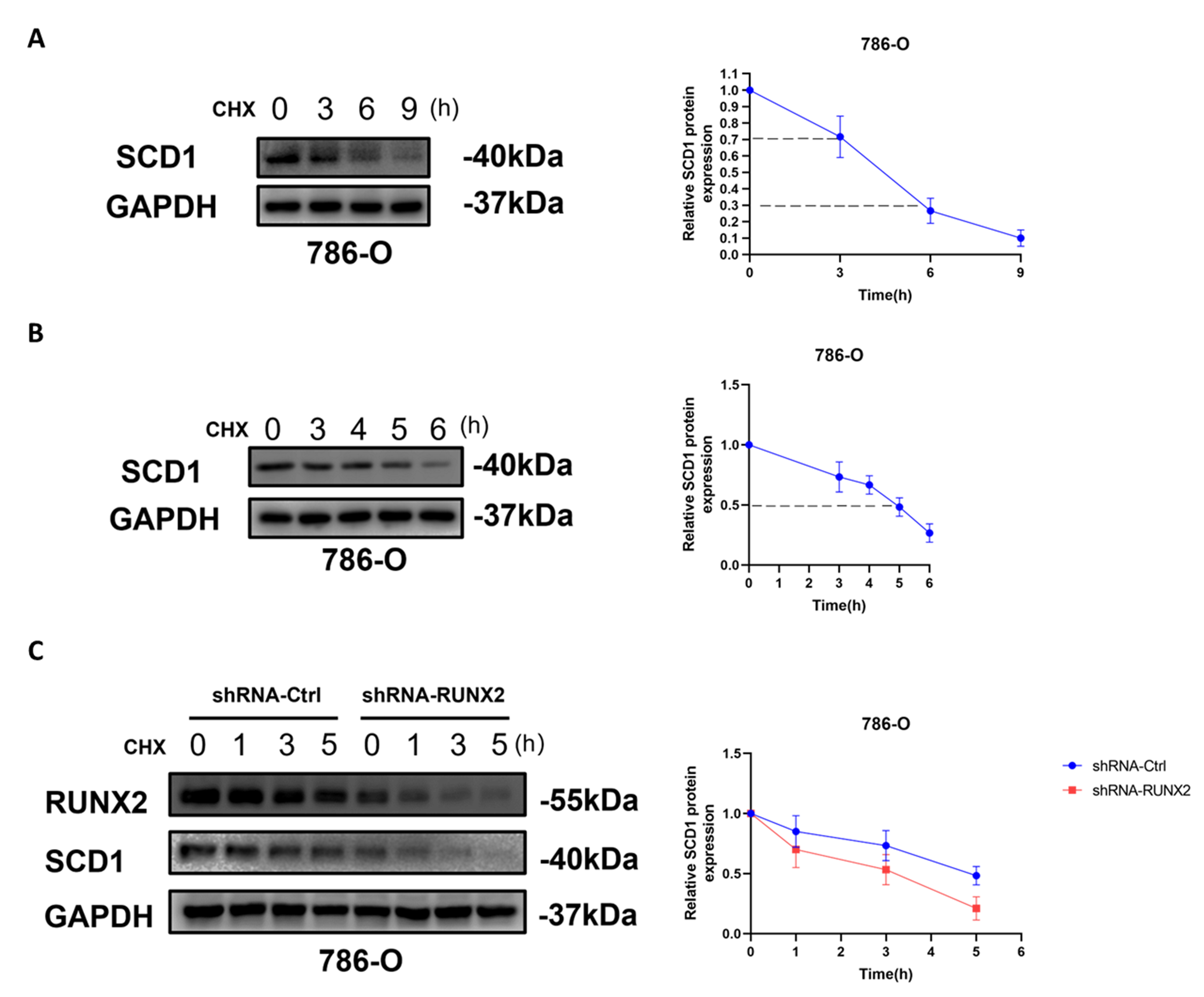

Supplement: Supplementary file 2 — Figure S2 [file CAM4-12-5764-s002.tif]
